# Feature enhancement guided network for yield estimation of high-density jujube

**DOI:** 10.1186/s13007-023-01066-2

**Published:** 2023-08-16

**Authors:** Fengna Cheng, Juntao Wei, Shengqin Jiang, Qing Chen, Yu Ru, Hongping Zhou

**Affiliations:** 1 College of Energy and Power Engineering, Nanjing Forestry University, Nanjing, 210037 China; 2 School of Computer, Nanjing University of Information Science and Technology, Nanjing, 210044 China

**Keywords:** Convolutional neural network, Regression, Object counting, Density map, Feature enhancement

## Abstract

**Background:**

Automatic and precise jujube yield prediction is important for the management of orchards and the allocation of resources. Traditional yield prediction techniques are based on object detection, which predicts a box to achieve target statistics, but are often used in sparse target settings. Those techniques, however, are challenging to use in real-world situations with particularly dense jujubes. The box labeling is labor- and time-intensive, and the robustness of the system is adversely impacted by severe occlusions. Therefore, there is an urgent need to develop a robust method for predicting jujube yield based on images. But in addition to the extreme occlusions, it is also challenging due to varying scales, complex backgrounds, and illumination variations.

**Results:**

In this work, we developed a simple and effective feature enhancement guided network for yield estimation of high-density jujube. It has two key designs: Firstly, we proposed a novel label representation method based on uniform distribution, which provides a better characterization of object appearance compared to the Gaussian-kernel-based method. This new method is simpler to implement and has shown greater success. Secondly, we introduced a feature enhancement guided network for jujube counting, comprising three main components: backbone, density regression module, and feature enhancement module. The feature enhancement module plays a crucial role in perceiving the target of interest effectively and guiding the density regression module to make accurate predictions. Notably, our method takes advantage of this module to improve the overall performance of our network. To validate the effectiveness of our method, we conducted experiments on a collected dataset consisting of 692 images containing a total of 40,344 jujubes. The results demonstrate the high accuracy of our method in estimating the number of jujubes, with a mean absolute error (MAE) of 9.62 and a mean squared error (MSE) of 22.47. Importantly, our method outperforms other state-of-the-art methods by a significant margin, highlighting its superiority in jujube yield estimation.

**Conclusions:**

The proposed method provides an efficient image-based technique for predicting the yield of jujubes. The study will advance the application of artificial intelligence for high-density target recognition in agriculture and forestry. By leveraging this technique, we aim to enhance the level of planting automation and optimize resource allocation.

## Background

Jujube tree, whose fruit is known as red jujube or Chinese jujube (date), is one of the world’s oldest cultivated fruit trees and the most important species in the wide international Rhamnaceae family in terms of economic, ecological, and social value [1, 2]. Jujube planting is a labor-intensive sector that needs a significant number of workers to complete numerous operations like planting, cultivation, and harvesting. Orchard production has increased dramatically in recent years as a result of the advancement of modern agriculture, and people are becoming increasingly interested in automatic management [3–5]. The yield estimation of jujube can assist managers in forecasting the output of the area of interest and enable producers to make appropriate professional modifications to the resource allocation of the orchard, which plays an important role in promoting the orchard’s benign development.

**Fig. 1:**
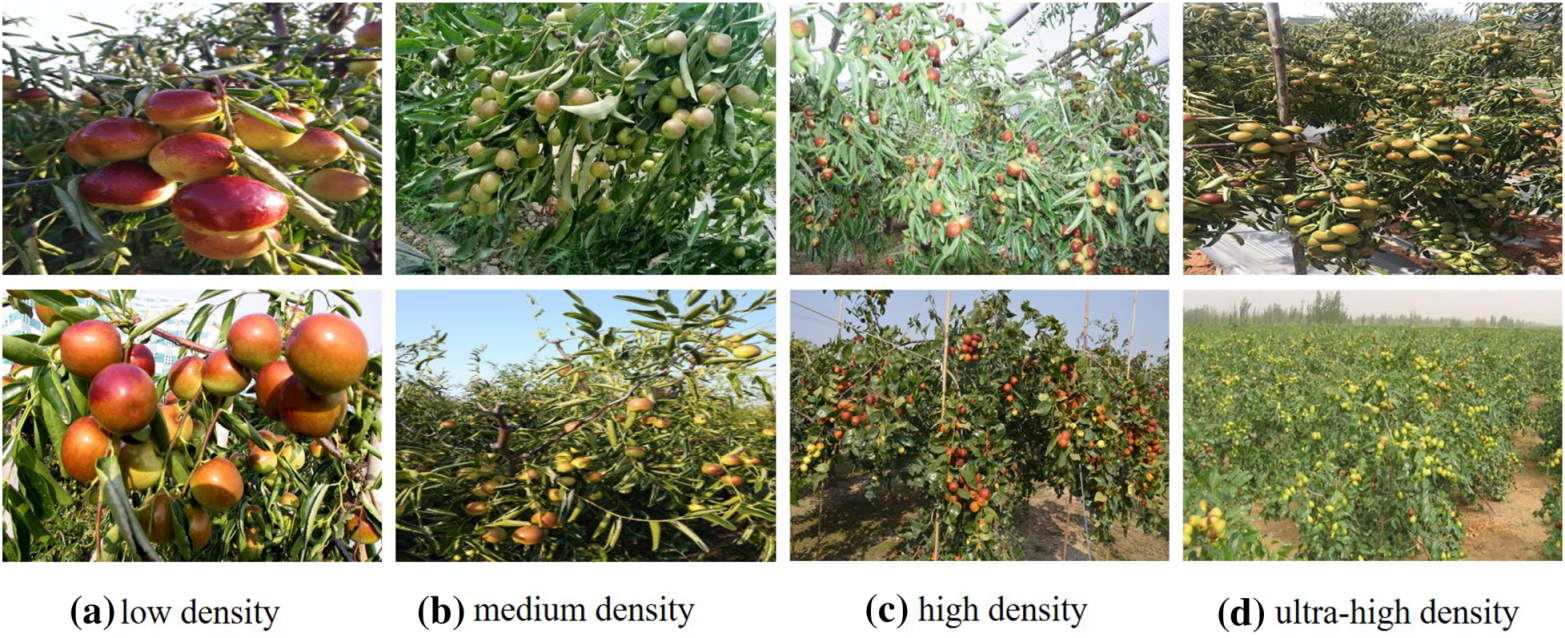
Part of samples with different densities from jujube counting dataset

However, this task is quite tough because of the high density, which makes it impossible for us to capture jujubes one by one. As an illustration, Fig. 1 depicts some samples of jujube taken from real scenes, which clearly indicates that the interested targets are subject to a variety of scales and severe occlusions. Additionally, the scene is complicated due to factors like changing illumination. According to modern cognitive research, human cognitive capacity is not a simple image-based matching process. It is an inherent process of information decomposition and reconstruction. Biological systems identify object features and draw conclusions about the unknown based on the learned features [6]. Based on this, computer vision algorithms offer a practical solution to the aforementioned issues.

Initially, traditional methods rely on the shape, color, or hand-crafted features to capture those interested objects [7]. Ref.[8] explored three prominent visual cues of texture, color, and shape into a strong classifier, which was used to capture berries even when they are of similar color to the vine leaves. These methods have a limited ability to generalize. When the camera angle of view or lighting conditions change, the prediction performance suffers greatly. To address these issues, several efforts are devoted to to building stronger representations [9, 10]. Ref. [11] proposed a multi-scale feature learning method for multi-class segmentation which is used to estimate the fruit yield on treecrops. Ref. [12] utilized a conditional random field on multi-spectral images (color and near-infrared reflectance) to model crop and background for the detection of highly occluded objects. Ref. [13] aggregated more robust feature representations for building an image descriptor, such as scale-invariant feature transform for calculating low-level features, bag of features. Although some issues have been improved [14], the applicability and robustness of those traditional models remain limited.

Inspired by the cognitive mechanism of biological natural vision, convolutional neural networks have been extensively developed [15–17]. Thanks to the development of deep learning in the field of computer vision [18–20], the performance on many tasks has been significantly improved. As for the task of jujube counting, an intuitive solution is to use detection-based methods. Many efforts have been made in this field thus far, with numerous milestone results, such as Faster-RCNN [21], FPN [22], YOLO and its variants [6, 23–26]. These efforts and follow-up detection work have promoted the detection and counting tasks in various parts of plants [27]. For example, [28] explored the use of an object detection framework, Faster R-CNN, in the context of fruit detection in orchards, such as mangoes and almonds. [29] trained four Region-based Convolutional Neural Networks (R-CNN) which output the spike density and a classification probability for each plot. The authors [30] used Faster-RCNN to detect the apple flowers, and the results were used to estimate the blooming intensity and determine the blooming peak date. Ref. [31] explored the data augmentation to increase the dataset size for detecting and counting olive fruit flies by using modified YOLO. Ref. [32] built a channel pruned YOLO model for accurately detecting apple fruitlets with a small model size. Afonso et al. [33] proposed a tomato detection and counting method based on Mask-RCNN. It performs well for recognizing and counting mature tomatoes, but it struggles to detect green fruits. Nevertheless, these methods are designed for scenes with relatively sparse targets, and their accuracy will suffer greatly when confronted with high-density scenes.

An alternative solution to the aforementioned problem is to regress the number of targets or the density map directly. MCNN [34] constructed a multi-column convolution network based on convolution kernels of different sizes to learn the scale change of targets, and they proposed a density map based on Gaussian kernels to characterize the number and distribution of targets. CSRNet [35] learned the multi-scale features of targets via the stack of dilated convolution layers, which achieves outstanding performance. MbCNN [36] perceived the scale change of the target by collecting the characteristics of different levels for aphid counting. These methods have produced good results, but their straight application to this task is not satisfactory because the distribution of targets is substantially different, as are the target density and size.

To address these issues, we propose a feature enhancement guided network for yield estimation of high-density jujube. To achieve this task, we collected a dataset with samples of different densities for jujube counting, which contains various challenges, such as severe occlusion, scale change, complex background, etc. Then, we put forward a new label generation method based on uniform distribution to characterize the jujube with point annotation. In addition, we designed a feature enhancement guided network for efficiently estimating the number of high-density jujubes. It consists of three main components: backbone, density regression module and feature enhanced module. The backbone is used to mine effective features from images, and the density regression module is given to predict the density map. To enhance the feature representation, a feature enhancement module is proposed to guide the density prediction with pixel-level semantic information. At last, various experiments are performed to demonstrate the effectiveness of our method. Our method achieves a more accurate estimation result with 9.62 MAE and 22.47 MSE compared to other state-of-the-art methods.

## Materials and methods

**Fig. 2:**
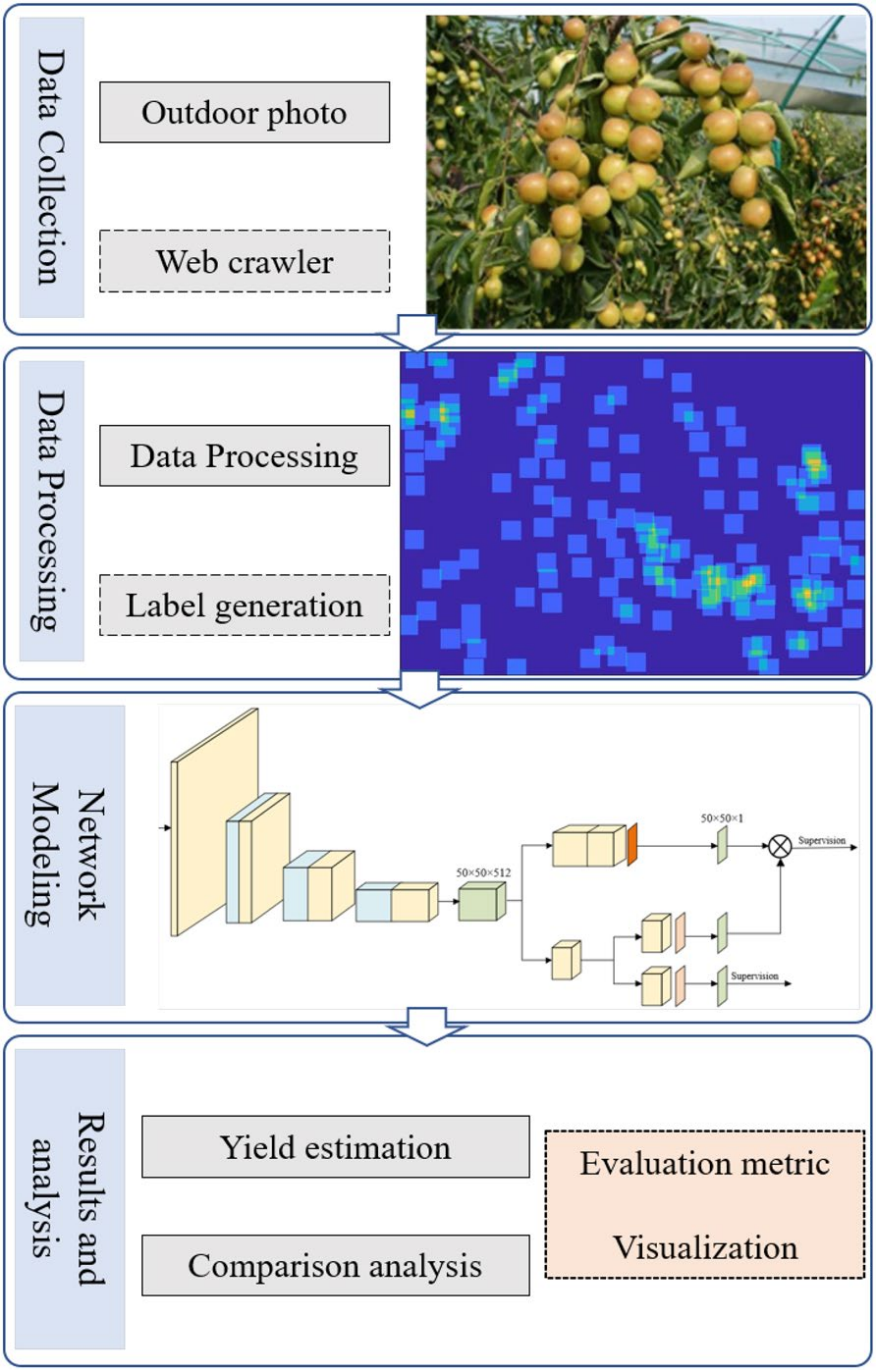
System overview

### Overall

The overview of a jujube yield estimation system is shown in Fig. 2. The main steps are as follows: The first is data collection, which can be performed using mobile phones, digital cameras, or web searching; the second is data processing, which includes data cutting, scaling, etc., to meet the computing abilities of hardware devices while creating labels for performance evaluation if necessary. The third is network modeling, wherein network reasoning can be performed directly on the processed data if the model has been trained. Note that pre-trained parameters are directly loaded for network reasoning. Finally, the results and analysis are given.

**Fig. 3:**
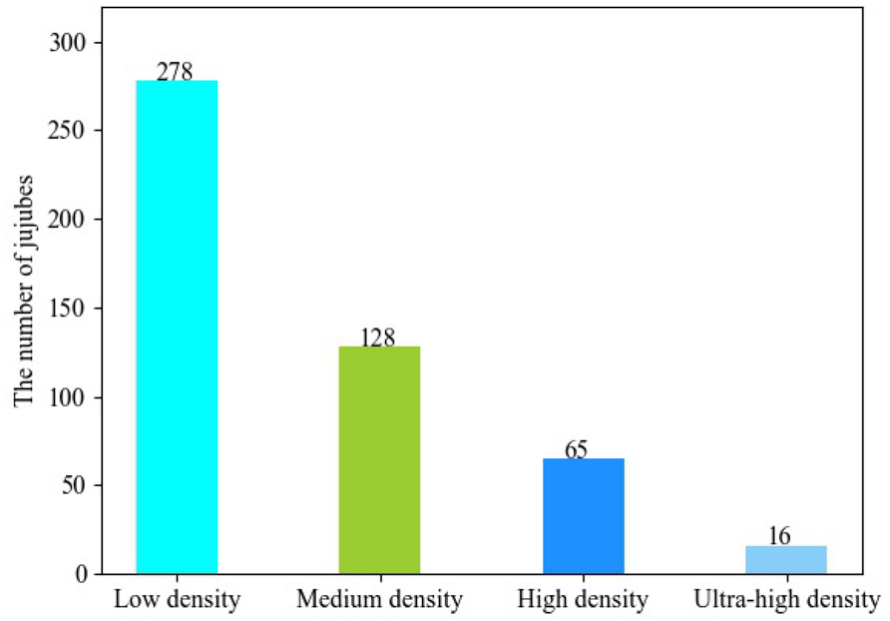
Distribution of samples at different densities

### Data collection

In this study, we collected a dataset for jujube counting with 692 images, which were primarily sourced from outdoor photos and web crawlers. It includes a range of types of jujubes, including winter jujube and pear jujube, to broaden the applicability of this dataset.

**Fig. 4:**
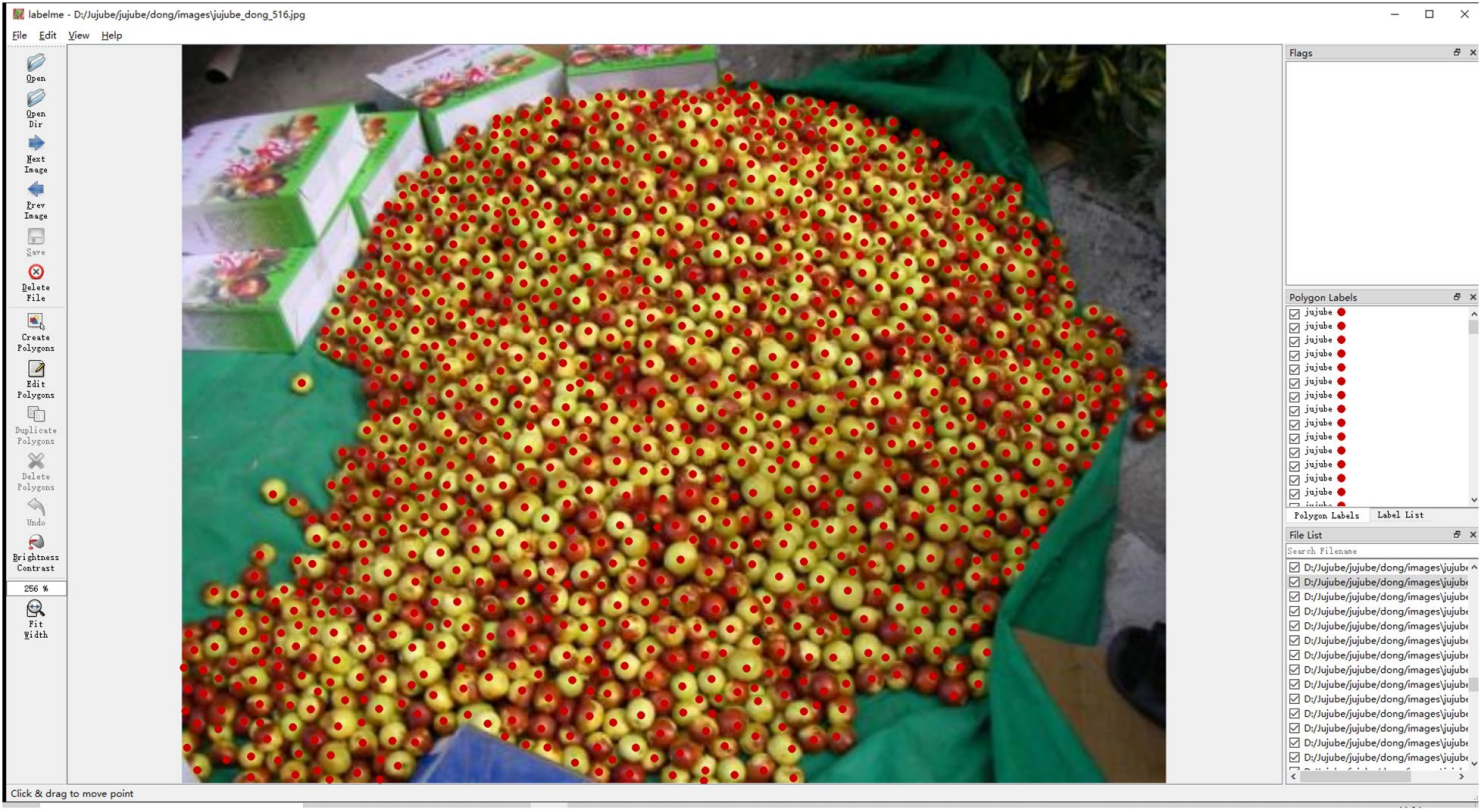
Image annotation. Red dots indicate labels

It covers samples with different densities. We separated them into four types for simplicity: low density (1–50), medium density (51–100), high density (101–200), and ultra-high density (more than 200), as shown in Fig. 1. The figure makes it clear that, compared to object detection in sparse scenes, this task is more challenging due to factors like severe occlusion, scale changes, and complicated background. With an average of 58 jujubes per image, we marked 40,344 jujubes in total. Additionally, Fig. 3 presents the distribution of samples at different densities. The dataset is divided into three parts: training, validation and test. In training phase, 459 and 50 samples are used for training and validation, respectively, and the rest are for test.

### Label generation

As shown in Fig. 1, in many scenes, the jujubes are small in scale and occluded more severely, which makes box annotation extremely difficult. For this reason, this work uses points to reduce the complexity of annotation. We use the publicly available annotation software named Labelme for annotation. A red dot at the center of each target indicates the position of the target (*x*, *y*), which represents the horizontal and vertical coordinates of the target, respectively. The labeling process is given in Fig 4, which shows a scene with a high density of jujubes, for which it obviously takes a long time to label.

Directly predicting points is a quite challenging task. To lessen the prediction complexity, we regress density map as an alternative. A common generation method is to generate the map based on Gaussian kernel, such as MCNN [34] and CSRNet [35]. Its purpose is to simulate objects of interest with various postures and perspectives. Instead, we introduce a new generation method of uniform distribution density map since the surface distribution of jujube is relatively uniform, which is easier to use and more efficient compared to the Gaussian-kernel based method. Assume that there is a jujube in position $x_i$ of the image. Then the uniform density map is generated by $\alpha (x-x_i)$. Formally, the overall density map is given by the following equation:

1 $$\begin{aligned} K = \sum \limits _{i = 1}^N \alpha (x - {x_i}), \end{aligned}$$

where *N* is the total number of the jujubes and $\alpha (x-x_i) = 1/M$ in which *M* is the area of density map.

**Fig. 5:**
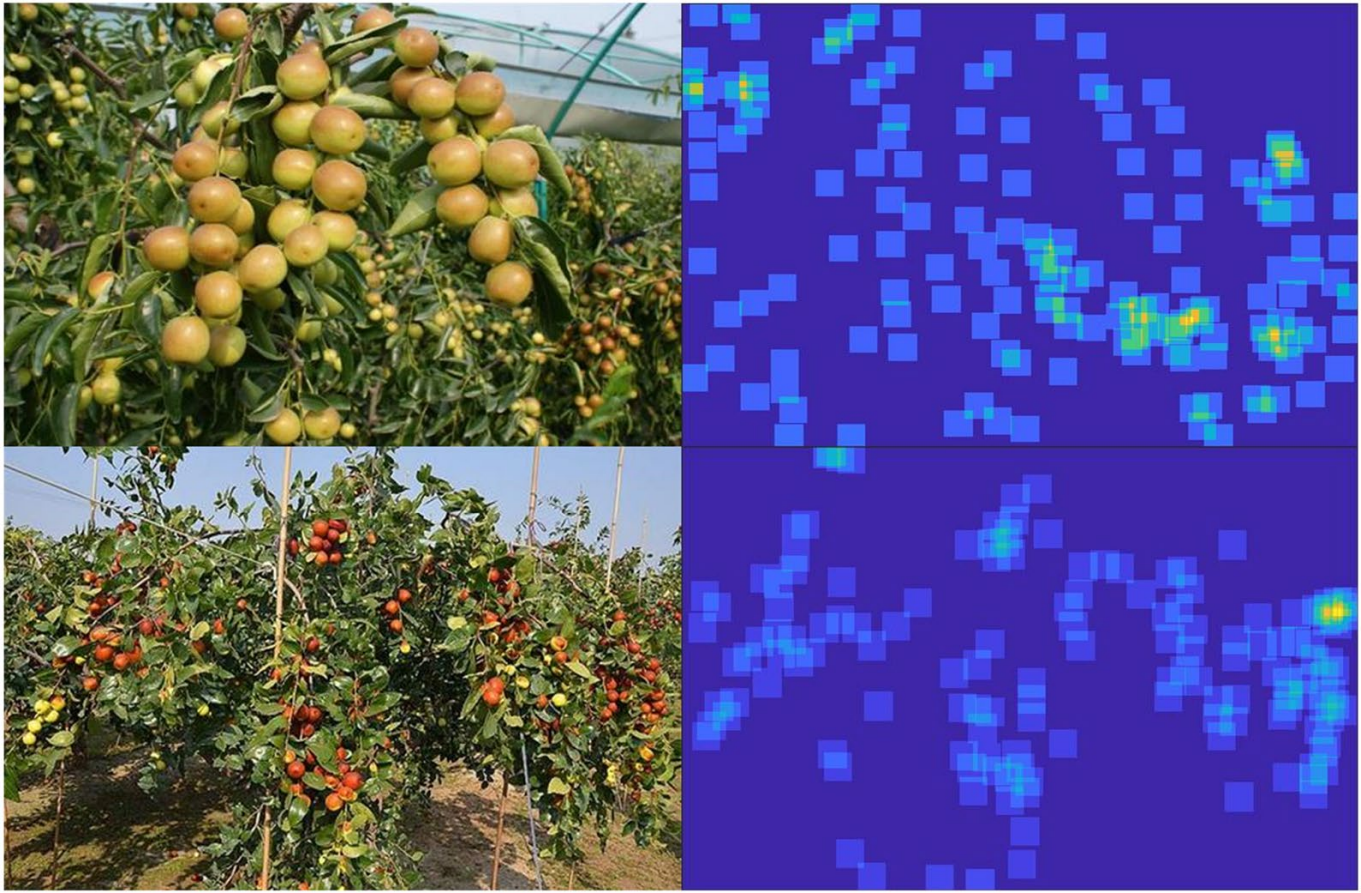
Visualization of the density map with uniform distribution

Figure 5 shows the visualization of the density map. It can be seen that it not only represents the quantity information of the targets, but also shows the distribution of the targets in the scene. This can help us sort out the local and global yield of the scenes.

### Feature enhancement guided network

**Fig. 6:**
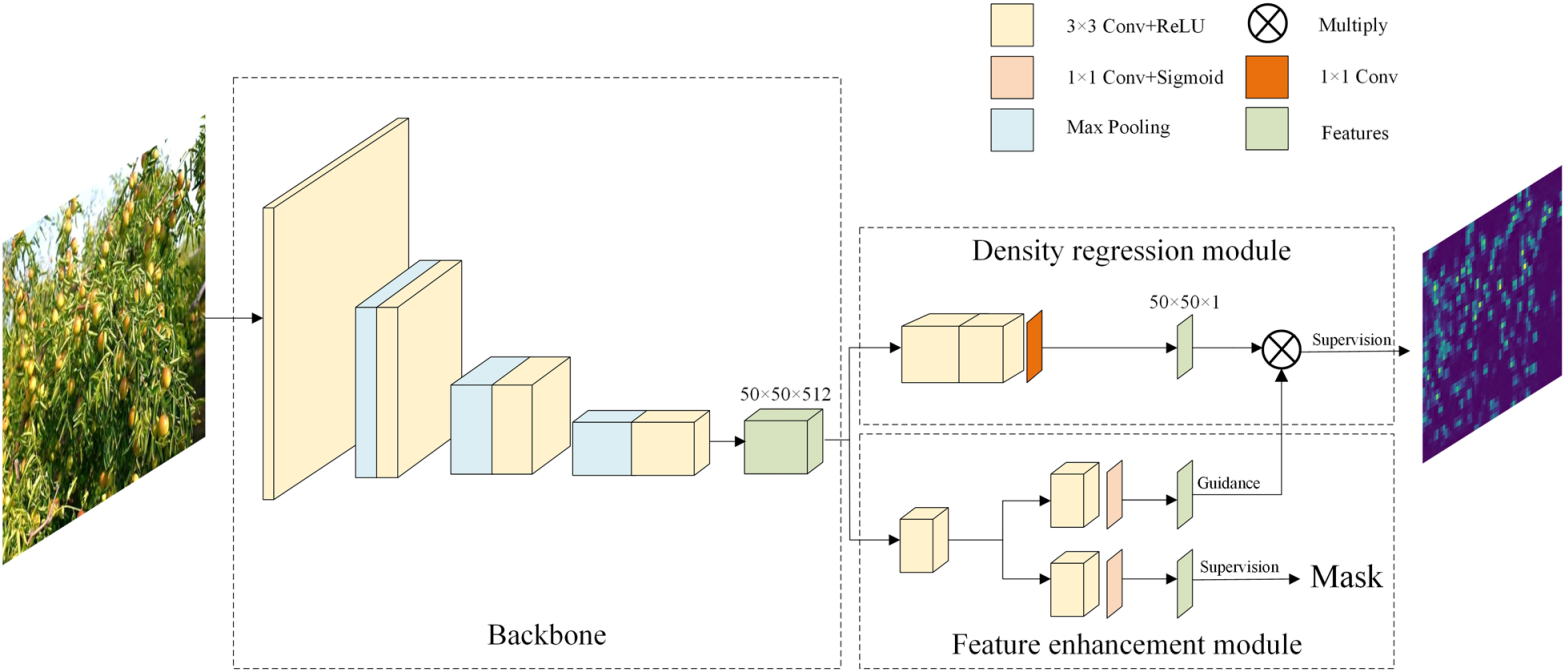
The overall framework of our proposed feature enhancement guided network. It is made up of three modules:backbone, density regression module and feature enhancement module

This study uses a convolutional neural network to extract robust features for jujube counting. Many networks, including MCNN, CSRNet, and MbCNN [36], have been proposed for density map regression. MCNN put forward three branches with various convolution kernels to achieve the sense of human head size. By using dilated convolution kernels and pre-trained VGG, CSRNet was able to learn about various scales. MbCNN performed feature enhancement by combining features at many levels. It is obvious from these findings that robust feature extraction is crucial for determining network performance. Inspired by these works, we propose a feature enhancement guided network (FEG Net) which aims to better direct the exploration of density maps, as shown in Fig. 6. It consists of three modules: backbone, density regression module and feature enhancement module, which will be introduced in detail.

#### Backbone

The backbone of neural networks is utilized to extract features from images, which is critical for the realization of robust vision tasks. To this end, we employ the first ten convolution layers of VGG-16 [18] as the feature extractor of our network. It should be emphasized that the network parameters pre-trained on ImageNet are loaded when the backbone is initialized. It aims to learn transferable prior knowledge, reduce the size of new task training data, and effectively avoid network overfitting.

The specific network configuration of the backbone is detailed in Table 1. More specifically, the first two layers are stacked by $3 \times 3$ convolution layers for extracting primitive information such as lines and corners. Following them are three pooling layers, each of which is followed by 2–3 convolution layers. Since three pooling layers are employed, we acquire a network feature that is 1/8 the size of the original image. It should be noted that each convolution layer is followed by a ReLU nonlinear transformation. As the number of network layers rises, the extracted features become more abstract, making it simpler for the network to detect concrete objects.

**Table 1:** Network configurations of the first ten layers of VGG16

First ten layers of VGG16	
Input images with three channels (RGB)	
conv3-64	
conv3-64	
maxpool	
conv3-128	
conv3-128	
maxpool	
conv3-256	
conv3-256	
conv3-256	
maxpool	
conv3-512	
conv3-512	
conv3-512	

#### Density regression module

The scale variation of jujubes is a remarkable challenge for this task. As depicted in Fig.  1, jujube size and shape vary widely due to the impact of the camera view. Inspired by [35, 37], this study utilizes dilated convolution to capture scale changes. This is because it increases the receptive field of the convolution kernel by a dilated stride without increasing parameters and avoids the spatial downsampling operation of the pooling layer. As a result, it enables flexible aggregation of multi-scale contextual features while retaining the same resolution.

In particular, we build four layers of $3 \times 3$ convolution with an expansion rate of two in this module, and the number of channels steadily decreases, which is 512, 512, 256, and 128 in turn. Finally, a $1 \times 1$ convolution layer is used to generate the final result ${O_d}({x_i})$. Due to the fact that the final output size is 1/8 of the original picture size, we further employ bilinear interpolation to restore the resolution to the original image size. The output of this branch accomplishes the network parameter optimization via the following loss:

2 $$\begin{aligned} Los{s_1} = 1 - {L_{x,y}}{C_{x,y}}{S_{x,y}}, \end{aligned}$$

where ${L_{x,y}} = \frac{{2{u_x}{u_y} + {c_1}}}{{u_x^2 + u_y^2 + {c_1}}}$, ${C_{x,y}} = \frac{{2{\sigma _x}{\sigma _y} + {c_2}}}{{\sigma _x^2\sigma _y^2 + {c_2}}}$, and ${S_{x,y}} = \frac{{{\sigma _{xy}} + {c_2}}}{{{\sigma _x}{\sigma _y} + {c_3}}}$ denotes luminance comparison, contrast comparison and structure comparison, respectively, ${u_m}$, ${\sigma _m}$ and ${\sigma _{mn}}$ denotes local mean, variance and covariance for predicted density map and groundtruth, respectively.

#### Feature enhancement module

This module builds dual paths as a guide for refining the features of the density regression module using supervised and unsupervised methods. The motivation of the module design is to emphasize the understanding of the region of interest and reduce the interference of noise. The reasons for designing dual paths are that there will be some semantic ambiguity if the output with the supervision signal is immediately filtered to the density regression module. As a result, we build two pathways to ensure that the module not only benefits from the semantic information provided by the supervision signal but also automatically adjusts the region of interest to improve network feature learning.

**Fig. 7:**
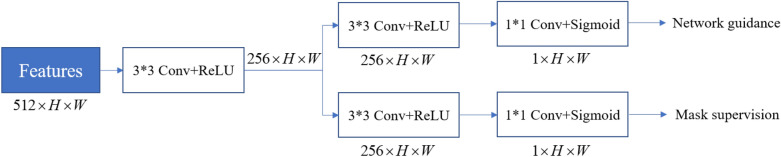
Feature enhancement module

The detailed configuration of this module is presented in Fig. 7. More specifically, the dual paths originate from a shared convolution layer, where each path has the same convolution structure. The shared convolution layer is made up of a $3 \times 3$ convolution layer with an expansion rate of 2. Each path is made up of a $3 \times 3$ convolution and a $1 \times 1$ convolution, followed by a sigmoid nonlinear operation. The supervised signals are given by a binary cross entropy loss:

3 $$\begin{aligned} Los{s_2} = \frac{1}{N}\sum \limits _i { - [{y_i}\log ({p_i}) + (1 - {y_i})\log (1 - {p_i})]}, \end{aligned}$$

where $p_i$ is the predicted probability value of each pixel, and $y_i$ is the binarization of density map by a given threshold.

The non-supervised path is used directly to filter the output of density regression module. The specific operation is listed as below:

4 $$\begin{aligned} De{n_f}({x_i}) = {O_d}({x_i})f({x_i}). \end{aligned}$$

where $f({x_i})$ denotes the output of the non-supervised path.

Finally, the parameters of the network are optimized as follows:

5 $$\begin{aligned} {L_{total}} = Los{s_1} + Los{s_2}. \end{aligned}$$

### Experiment settings

The proposed network is built based on the Pytorch framework, and we generate the density map by MATLAB. Unified hardware is used for training and test, namely Intel Core i7-11700K, 16GB RAM and RTX 2080Ti. We train our model for 600 epochs with batch size 8. Adam optimizer is used to optimize the network, and the learning rate is $10^{-5}$. In the training process, we use random clipping, random gamma transform, random grayscale and random flip for data augmentation.

### Evaluation metrics

We use Mean Absolute Error (MAE) and Mean Square Error (MSE) as evaluation metrics to evaluate our model which is defined in the following:

6 $$\begin{aligned} MAE = \frac{1}{N}\sum _{i=1}^{N}|Z_i-\hat{Z}_i| \end{aligned}$$

and

7 $$\begin{aligned} MSE = \frac{1}{N}\sum _{i=1}^{N}(Z_i-\hat{Z}_i)^{2} \end{aligned}$$

where *N* is the number of test images, $Z_i$ is the actual number of jujubes in the *i*th image, $\hat{Z}_i$ is the estimated number of jujubes in the *i*th image. Note that MAE is used to evaluate the average accuracy of network prediction, while MSE is used to evaluate the robustness of the proposed network.

## Results and analysis

In this section, we study the influence of network structure and settings on counting performance. Comparative experiments are finally provided to demonstrate the effectiveness of our proposed network.

### Comparison results on feature enhancement module

We perform an ablation study on the feature enhancement module. To more clearly illustrate the benefits of this module, we will initially provide three baselines as follows.

- Baseline 1: It is a variant of CSRNet (termed as CSRNet$^*$), consisting of four $3 \times 3$ convolutions with an expansion rate of 2, followed by a $1 \times 1$ convolution.
- Baseline 2: It has two $3 \times 3$ convolutions with an expansion rate of 2, followed by a $1 \times 1$ convolution and Sigmoid activation function. To generate the final density map, multiply the output of the aforementioned operations by that of Baseline 1.
- Baseline 3: It has the same network structure with Baseline 2. The distinction is that there is no multiplication operation taking place here. As an auxiliary signal, the mask is utilized as the supervised signal to efficiently help the network better distinguish between the foreground and background.

Clearly, baseline 2 and baseline 3 are one of the two paths in our module. The reason for setting these two baselines is to emphasize the benefits of combining the two in our module. To make this ablation study more applicable, we adopt the same settings as CSRNet, such as the parameter initialization of pre-trained VGG, and label generation. Table 2 presents the comparison results of our proposed module with the three baselines. Compared to baseline 1, baseline 2 shows a slight improvement in MAE performance while simultaneously significantly reducing MSE. This outcome can be attributed to the use of unsupervised attention, which directly constructs a single path to focus on learning regions of interest. However, due to the presence of noise, efficient identification of both interesting and uninteresting regions becomes challenging. In comparison to both baseline 1 and baseline 2, baseline 3 demonstrates a significant improvement in both MAE and MSE. This suggests that the utilization of mask-based supervised signals enables better perception of important regions. Nevertheless, the generation of masks based on a given threshold introduces a certain amount of noise. To address these challenges, we propose a module that combines the strengths of both approaches. By doing so, we are able to further mitigate the impact of noise and consistently filter out unimportant regions more effectively.

**Table 2:** Comparison results of our proposed module with different baselines

Model	MAE	MSE
Baseline 1	13.37	29.81
Baseline 2	12.66	32.38
Baseline 3	11.30	26.03
FEG Net$^*$	10.76	23.32

FEG Net $^*$ represents FEG Net with MSE loss as the supervisory signal

### Comparison results on loss function

In order to properly train the network, the quality of loss function is crucial. It can make the network’s output adhere more closely to the actual distribution of labels. In this subsection, we evaluate the impact of different loss functions on network performance. Table 3 presents the comparison results of different loss functions. In analyzing the table, it becomes evident that the performance of the L1 loss is poor. This can be attributed to the non-differentiability of the loss function at zero, resulting in slow convergence rates of the optimization algorithm. Consequently, fitting the density map becomes more challenging. On the other hand, MSE loss exhibits improved performance by effectively fitting the density map. However, it is susceptible to outliers, meaning that large discrepancies between predicted and true values can lead to gradient explosion, adversely affecting network parameter optimization. To address these issues, the Smooth L1 loss is proposed as a suitable alternative. This loss function effectively avoids the aforementioned problems and demonstrates improved performance in both metrics, as evidenced by the table. Additionally, the SSIM loss prioritizes differences between structures rather than pixel-level discrepancies, thereby excelling in MAE performance. Consequently, in this study, we opt for SSIM loss as the objective for network optimization.

**Table 3:** Comparison results of different loss functions

Loss function	MAE	MSE
L1 loss	26.90	39.36
MSE loss	10.76	23.32
Smooth L1 loss	10.40	21.11
SSIM loss	9.69	21.78

### Comparison results on data augmentation

Due to the small amount of data for this task, we use various data augmentation techniques to increase the training samples so that the trained model has stronger generalization ability. Table 4 presents the experimental results with or without data augmentation. It clearly shows that the training method with data augmentation can greatly enhance the network performance in this task, with MAE increased by about 19.4% and MSE increased by 9.1%..

**Table 4:** Comparison results of the proposed network with/without data augmentation

Data augmentation	MAE	MSE
Without augmentation	12.02	23.96
With augmentation	9.69	21.78

### Comparison results on label generation

Due to the diverse characteristics of targets, general counting tasks typically use Gaussian kernels to describe the distribution of targets. This distribution may enable the network to more effectively capture the apparent characteristics of targets. Since the distribution of the targets in this study is relatively uniform, with the exception of color, we use the uniform distribution representation. Table 5 makes it clear that this strategy can produce a better MAE performance with less effort. Simultaneously, we have observed that the difference between the two approaches is not significant. This could be attributed to the manual setting of the density map with a fixed size, which makes it challenging to accurately cover the target region. In other words, the generated maps for each point tend to be either too large or too small. The ablation study reveals two important findings. Firstly, the representation of the target in this task is not singular, indicating that selecting an appropriate representation can contribute to enhancing the performance of single or multiple metrics. Secondly, exploring the topic of adaptively setting the size of the density map to precisely cover the target area holds significant potential and warrants further investigation.

**Table 5:** Comparison results of the proposed method with different label generation methods

Method	MAE	MSE
Gaussian distribution	9.69	21.78
Uniform distribution	9.62	22.47

### Comparison results with the state-of-the-art models

In this subsection, we compare the proposed network with other networks designed for the counting task, including object detection-based methods (Faster RCNN and YOLO) and density regression-based methods (MCNN, CSRNet, and MbCNN). For object detection methods, we use point information to generate suitable pseudo-boxes and then use them to train these two object detection methods. MCNN built three convolutional branches with various scales and then combines them. Based on the pretrained VGG, CSRNet used dilation convolution to learn the scale change of the target. To improve the target’s characterization, MbCNN employed convolutional features with various hierarchical scales.

Table 6 shows the comparison results of the proposed method with these methods. Notably, the proposed network performs the best. The satisfactory results clearly confirm the effectiveness of our proposed strategies. The table clearly demonstrates that detection-based methods generally exhibit low MAE but high MSE. This is primarily because these methods excel at accurately detecting targets when they are large in size. However, they struggle to perform effective detection when the targets are small and heavily occluded. Moreover, the table highlights that YOLO outperforms Faster RCNN significantly. This improvement in performance can be attributed not only to the network structure itself but also to the incorporation of rich data augmentation strategies employed by YOLO. Furthermore, YOLO V5m demonstrates superior performance compared to YOLO V5s, achieving a MAE of 12.39 and a MSE of 41.95. This enhancement can be attributed to the increased number of parameters that enable more expressive features to be captured. Among the density regression-based methods, both MCNN and MbCNN are lightweight networks with relatively poorer performance. CSRNet utilizes pre-trained models to initialize its network parameters, enabling better capture of the target compared to MCNN and MbCNN. However, when compared to CSRNet, our proposed method showcases significant improvements, achieving a MAE of 9.62 and an MSE of 22.47. These advancements are primarily attributed to the effectiveness of our proposed module.

**Table 6:** Comparison of results between the proposed method and other counting methods

Method	MAE	MSE
Faster RCNN	15.78	46.20
YOLO V5s	13.29	43.66
YOLO V5m	12.39	41.95
MCNN	31.52	57.84
CSRNet$^*$	13.37	29.81
MbCNN	33.35	60.86
FEG Net	9.62	22.47

FEG Net uses SSIM loss as the supervisory signal

**Fig. 8:**
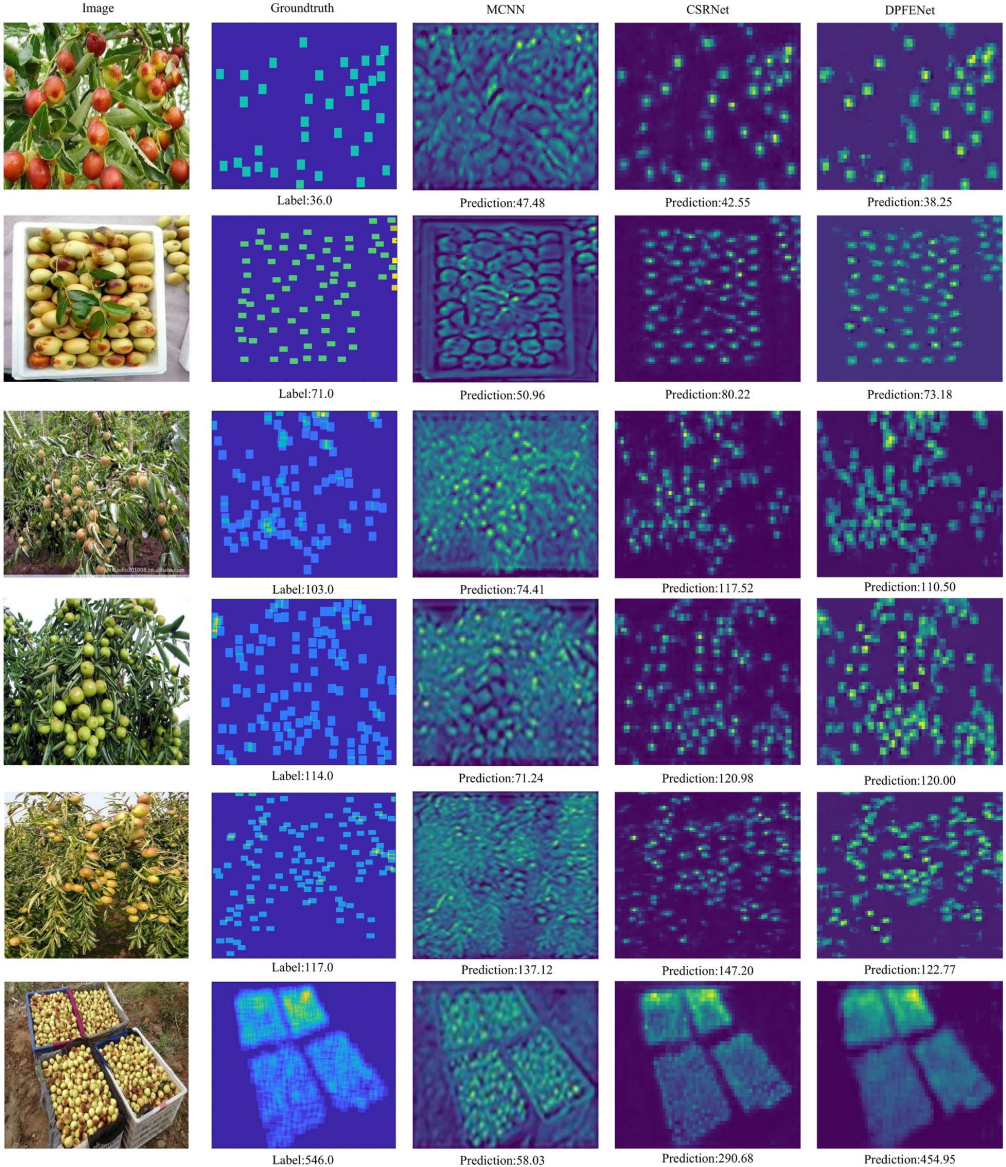
Comparison of the predicted results of our method with other methods

Figure 8 shows the comparison of predicted results between our method and other counting methods. It should be noted that the results include pictures from low density to high density. As shown, the number of jujubes produced by our network is closer to the groundtruth when compared to the other two networks. We also notice that the performance of the three networks at high density, such as the last line of the image, is not satisfactory, but our performance remains the best. Simultaneously, when compared to other networks, we generate less noises in the density map. Finally, it is not difficult to summarize the effectiveness of our proposed method.

## Conclusions

In this paper, we introduce a feature enhancement guided network for high-density jujube counting. To begin with, a new jujube counting dataset is collected, which covers most challenges in this task, including severe occlusion, scale change, and complex background. To better characterize the dot annotation, we then use uniform distribution to generate the image label in a density map fashion. Different from the Gaussian kernel-based method, the proposed method is easier and more effective. Next, a feature enhancement guided network is introduced to estimate the number of jujubes. It mainly consists of backbone, density regression module and feature enhanced module. The first two are used to extract features and predict the density map, respectively. The last one is a new proposed module to assist the density regression module in robust feature extraction. It uses mask signals as supervision to gain pixel-level semantic information to better characterize interested objects. At last, a number of experimental studies are provided to support the validity of our proposed approaches, and our network outperforms other state-of-the-art models.

## Data Availability

The datasets used and/or analyzed during the current study available from the corresponding author on reasonable request.
